# Decreased Expression of a Gene Caused by a T-DNA Insertion in an Adjacent Gene in *Arabidopsis*

**DOI:** 10.1371/journal.pone.0147911

**Published:** 2016-02-01

**Authors:** Kentaro Tamura, Takenori Kawabayashi, Toshiharu Shikanai, Ikuko Hara-Nishimura

**Affiliations:** Graduate School of Science, Kyoto University, Kyoto, Japan; Iwate University, JAPAN

## Abstract

ALADIN is a component of the nuclear pore complex in higher eukaryotes. An *Arabidopsis* knockout line that had a T-DNA insertion in the *ALADIN* gene was defective in plant growth and thylakoid development and had reduced photosynthetic activity resulting from lower chlorophyll accumulation. The mutation appeared to decrease the level of chloroplast RuBisCO subunits and PSBA and PGL35 proteins. Unexpectedly, the T-DNA insertion in the *ALADIN* gene decreased the expression of the neighboring gene *PSRP5*, which functions in translation in chloroplasts. The mutant phenotype was rescued by expressing *PSRP5*, but not by expressing *ALADIN*. The abnormal phenotypes were also detected in an artificial microRNA (amiRNA)-mediated *PSRPS5* knockdown, but not in an amiRNA-mediated *ALADIN* knockdown line. Thus, users of T-DNA insertions should be aware that a T-DNA insertion in one gene can have effects on the expression of neighboring genes.

## Introduction

Since the completion of the *Arabidopsis* genome sequence, a key aim of the plant research community has been to identify the function of each gene. One widely-used reverse genetic technique is insertional mutagenesis mediated by *Agrobacterium* transformation and T-DNA insertion [1]. T-DNA flanked by specific 25-bp direct repeats is transferred from *Agrobacterium* into plants [2]. A piece of T-DNA (more than 5-kbp fragment) is inserted randomly into genome, resulting in causing significant effects on the gene function. Depending on the insertion site and the nature of T-DNA, it leads several effects, such as knockout, knockdown, and knockon. Over the past decade, phenotypes associated with T-DNA insertions have played a critical role in advancing plant research. Large collections of *Arabidopsis* T-DNA insertion lines have been, and continue to be, developed around the world [3–7]. Approximately 88% of all *Arabidopsis* genes are thought to have been disrupted at least once [1]. Recently, by using a next-generation sequencing method, flanking sequences of 146,740 insertions were also identified (http://www.arabidopsis.org). T-DNA mutagenesis has been used in functional genomic analysis of species other than *Arabidopsis*, such as rice [8] and *Brachypodium* [9].

Despite its utility, T-DNA mutagenesis has several limitations. One disadvantage is that T-DNA integrations are often complex and can lead to the deletion or chromosomal rearrangement of the surrounding genomic DNA [10, 11]. This significantly complicates subsequent molecular analysis in the mutant. Here, we describe a T-DNA-associated mutation that unexpectedly affected the expression level of a neighboring gene in *Arabidopsis*, identified during the course of reverse genetic analysis of a nucleoporin.

## Materials and Methods

### Plant materials

*Arabidopsis thaliana* (ecotype Wassilewskija) was used as wild type. A T-DNA insertion mutant (FLAG_453B04) in the Ws background was obtained from the Versailles *Arabidopsis* Stock Centre at the Jean-Pierre Bourgin Institute of the National Institute for Agricultural Research (INRA).

### Transgenic plants

Genomic fragments containing either the *ALADIN* (At3g56900) gene or the *ALADIN* + *PSRP5* (At3g56910) genes were generated using specific primers (S1 Table) and cloned into pENTR1A (Invitrogen, USA). The *ALADIN* genomic fragment contains a region from 2-kbp upstream to 0.3-kbp downstream of the *ALADIN* coding sequence. The *ALADIN* + *PSRP5* genomic fragment contains a region form 0.13-kbp upstream of the *PSRP5* coding sequence to 2-kbp upstream of the *ALADIN* coding sequence. Cloned DNA fragments were transferred from the entry clone to the pFASTG01 Gateway destination vector [12] by an *in-vitro* recombination attL × attR reaction. *ALADIN* and *PSRP5*-directed artificial microRNA (amiRNA) constructs were designed using a Web-based program (http://wmd2.weigelworld.org) [13, 14]. Corresponding fragments were generated using specific primers (S1 Table) and were cloned into pENTR1A (Invitrogen). Cloned DNA fragments were then transferred from the entry clone to the pFASTG02 Gateway destination vector [12] by an *in-vitro* recombination attL × attR reaction.

### RT-PCR

Total RNA was isolated from 14-day-old plants using an RNeasy Plant Mini Kit (Qiagen, USA). Reverse transcription was performed using Ready-To-Go RT-PCR Beads (GE Healthcare, USA) with an oligo (dT)_12-18_ primer. Gene-specific primers are given in S1 Table. PCR products were visualized using agarose gel electrophoresis with EtBr.

### Measurement of chlorophyll content and the maximum quantum efficiency of photosystem II

Chlorophyll content was determined in mature first and second leaves of 14-day-old plants as described previously [15]. The maximum quantum efficiency of photosystem II was quantified using a Mini-PAM chlorophyll fluorometer (Walz) as described previously [16]. The difference between maximum chlorophyll fluorescence (Fm) and minimum chlorophyll fluorescence at the open photosystem II center is defined as variable fluorescence (Fv). The maximum quantum efficiency of photosystem II is indicated by Fv/Fm.

### Transmission electron microscopy

Mature leaves of 14-day-old plants were used for transmission electron microscopy. Samples were prepared as described previously [17]. Images were obtained using a transmission electron microscope (JEM-1200 EX; JEOL, Japan) at an acceleration voltage of 80 kV.

### SDS-PAGE and immunoblot analysis

Protein extracts from plants were subjected to SDS-PAGE followed by either Coomassie Brilliant Blue (CBB) staining or immunoblot analysis. Immunoreactive signals were detected using an ECL detection system (GE Healthcare) with the following antibodies: anti-RBCL (1:1000 dilution), anti-RBCS (1:1000), anti-PSBA (1:5000), and anti-PGL35 (1:1000) (Agrisera, Sweden).

### Statistics

Mean, standard deviation (S.D.), and two-tailed Student t-test calculations were performed using Microsoft Excel with StatPlus software.

## Results and Discussion

### An *aladin* knockout mutant is defective in photosynthesis

**Fig 1:**
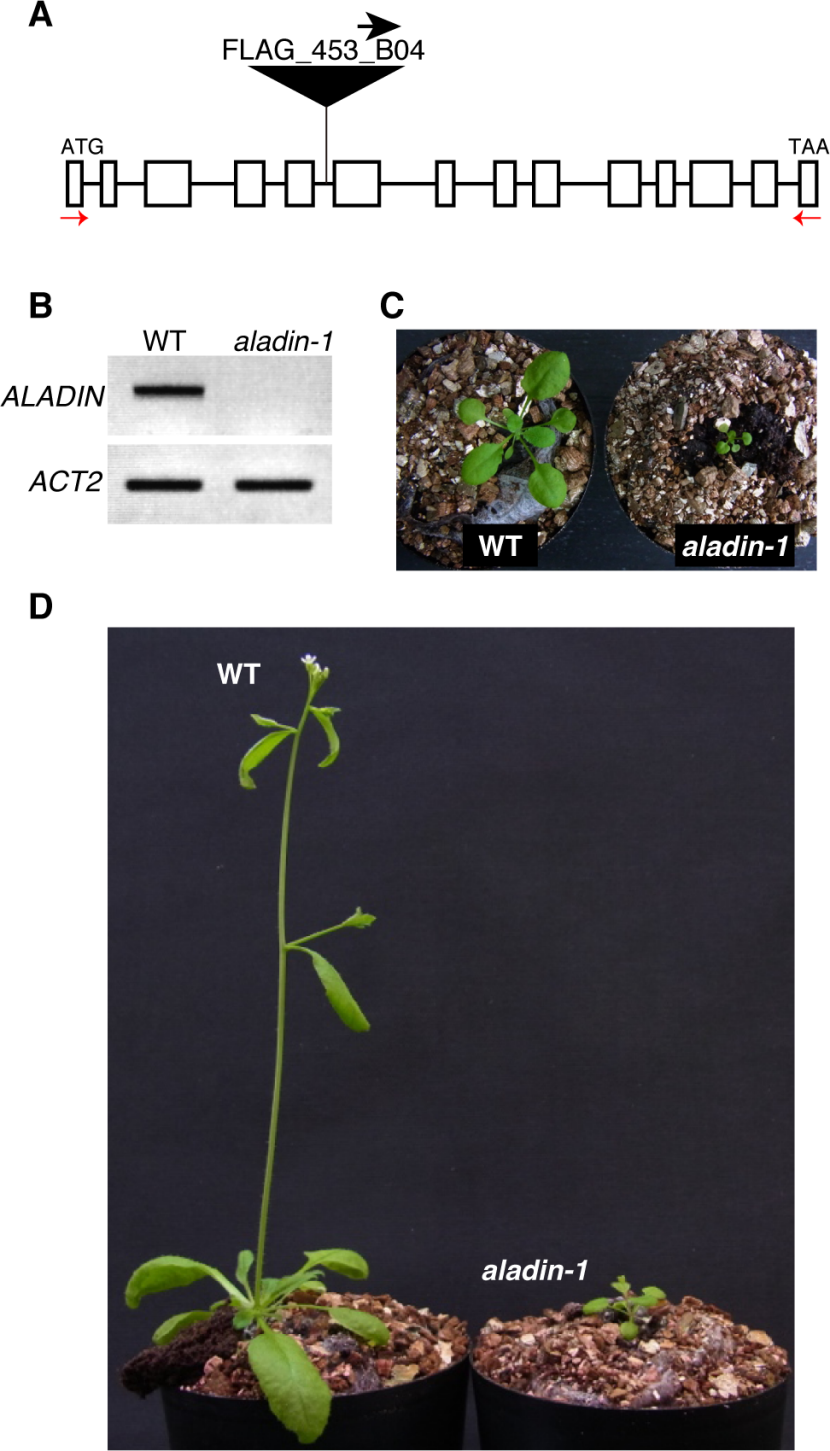
Isolation of the *aladin-1* mutant. (A) Schematic representation of the *ALADIN* gene. A T-DNA insertion site in the FLAG_453B04 line is shown. The orientation of the left border sequence is indicated by an arrow. (B) RT-PCR analysis of *ALADIN* transcription in the *aladin-1* mutant. *ACTIN2* (*ACT2*) was used as a loading control. (C-D), Three-week- (C) and five-week- (D) old wild-type (WT) and *aladin-1* plants.

ALADIN (ALacrima Achalasia aDrenal Insufficiency Neurologic disorder) is an evolutionally conserved nucleoporin that is a member of the nuclear pore complex (NPC) in higher animals and plants [18, 19]. In humans, mutations in the *ALADIN* gene lead to a rare autosomal recessive disorder called the triple A syndrome [20, 21]. However, mice lacking a functional *ALADIN* gene do not exhibit this phenotype and are indistinguishable phenotype from wild-type mice [22, 23], suggesting a species-specific function for ALADIN. In this study, a single line knockout mutant from the FLAG T-DNA Versailles INRA collection was used to examine the physiological role of ALADIN in *Arabidopsis* (Fig 1A). Another ALADIN T-DNA knockout mutant (SALK_148848 line) was available, but any plants had no T-DNA insertion in *ALADIN* gene. Genomic sequencing of the T-DNA flanking region confirmed that the *aladin-1* mutant (FLAG_453B04) had a T-DNA insertion in the middle of the *ALADIN* gene (S1 Fig). RT-PCR revealed that the mutant accumulated no full-length *ALADIN* transcript (Fig 1B) but a truncated transcript (S2 Fig).

**Fig 2:**
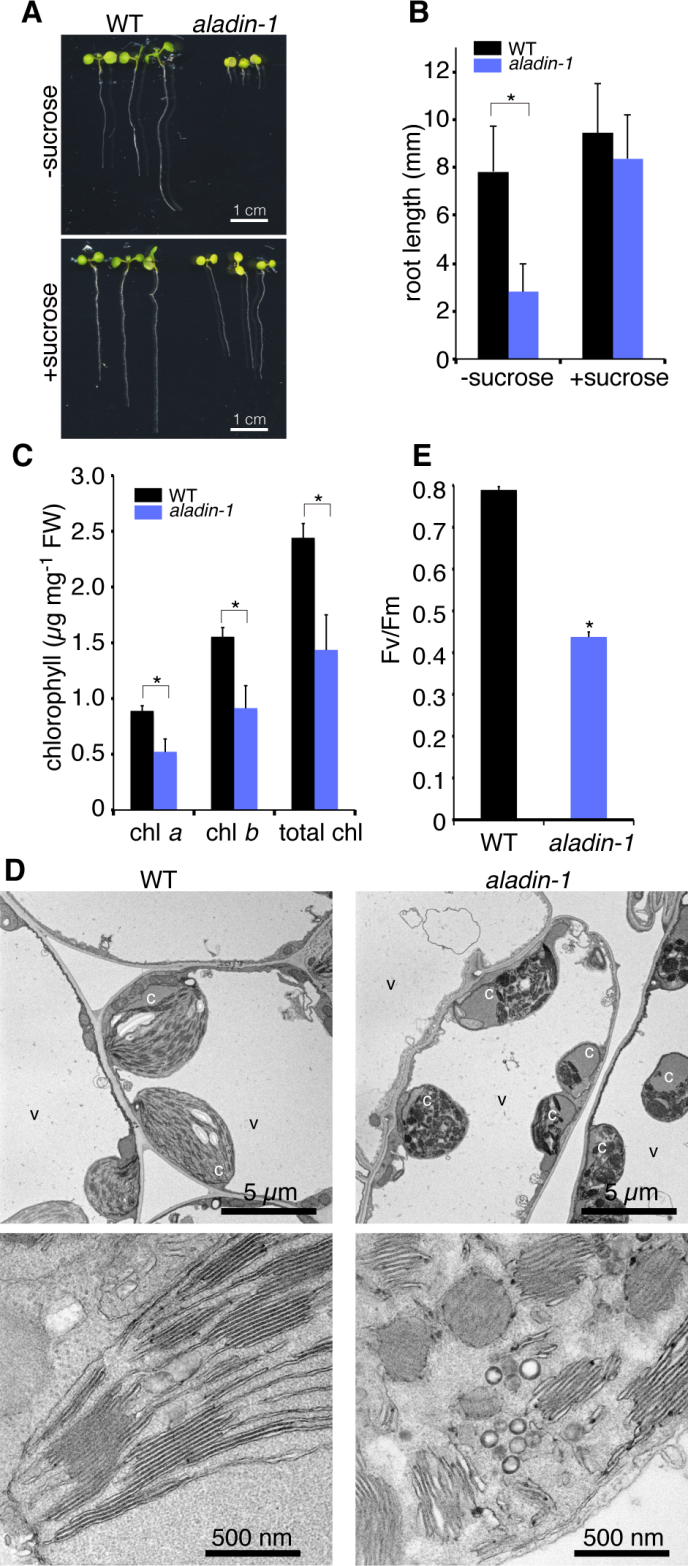
The *aladin-1* mutant exhibits low photosynthetic activity. (A) Seven-day-old wild-type (WT) and *aladin-1* seedlings grown on MS plates with (+ sucrose) or without (- sucrose) sucrose. (B) Root length of 10-day-old WT and *aladin-1* seedlings grown on MS plates with (+ sucrose) or without (- sucrose) sucrose. Mean ± standard deviation for n > 20 (Student’s t-test, *P < 0.001). (C) Quantification of chlorophyll *a* (chl *a*), chlorophyll *b* (chl *b*), and total chlorophyll (total chl) in mature WT and *aladin-1* leaves. Mean ± standard deviation for n > 3 (Student’s t-test, *P < 0.001). (D) Electron micrographs of mesophyll cells (upper) and chloroplasts (lower) in mature leaves from 14-day-old WT and *aladin-1* plants. c, chloroplast; v, vacuole. (E) Maximum quantum yield of photosysytem II (Fv/Fm).

The *aladin-1* mutant exhibited pleiotropic growth defects at various developmental stages. Compared to the wild type, the mutant showed extremely stunted growth (Fig 1C), including dwarfism (Fig 1D), and a shorter root length in seedlings (Fig 2A). The shorter root phenotype was partially rescued by exogenous sucrose (Fig 2A and 2B), suggesting an impairment of carbon metabolism in the mutant. Mutant leaves were pale green in color and contained less chlorophyll than wild type (Fig 2C). These results indicated that the *aladin-1* mutant was significantly impaired in chloroplast development and photosynthesis. Next, electron microscopic analysis was performed to examine the ultrastructure of the chloroplasts. Chloroplasts in epidermal cotyledon cells were smaller and had a more irregular shape in the mutant than in wild type (Fig 2D, upper panels). Furthermore, in mutant cells, thylakoid membranes were highly fragmented and vesicle-like structures were apparent (Fig 2D, lower panels). The maximum quantum efficiency of photosystem II was 55% lower in the mutant than in wild type (Fig 2E). These results indicated that the *aladin-1* mutation hindered the normal development of the thylakoid membrane and led to a loss of photosynthetic activity.

**Fig 3:**
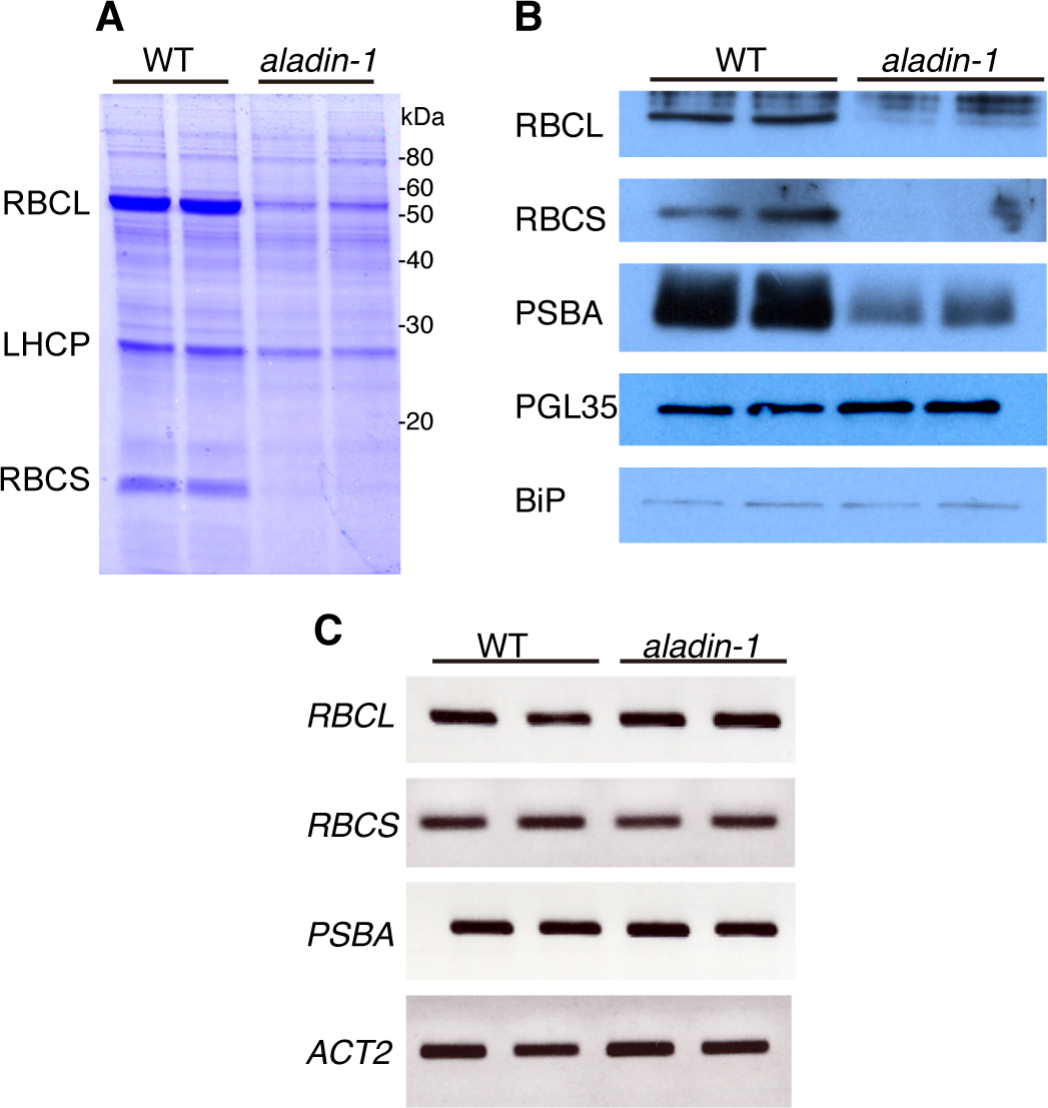
Translation of chloroplast proteins is significantly impaired in *aladin-1*. (A-B) Protein extracts from 7-day-old wild-type (WT) and *aladin-1* seedlings were subjected to SDS-PAGE followed by either Coomassie Brilliant Blue staining (A) or immunoblotting (B) with anti-RuBisCO large subunit (RBCL), RuBisCO small subunit (RBCS), light-harvesting chlorophyll a/b binding protein (LHCP), photosystem II reaction center protein A (PSBA), plastoglobulin 35 kDa (PGL35), or binding protein (BiP) antibodies. Molecular masses are indicated on Fig 3A (kDa). Technical duplicate was done: two independent extractions of protein (A) and mRNA (B) were subjected to two lanes on each panel. (C) RT-PCR analysis of *RBCL*, *RBCS*, *PSBA*, and *ACT2* transcription in WT and *aladin-1*. Technical duplicate was done: two independent extractions of mRNA were subjected to two lanes on each panel.

Accumulation of chloroplast proteins was investigated by SDS-PAGE and immunoblot of lysates from mature leaves. Accumulation of RuBisCO subunits and PSBA (photosystem II reaction center protein A) was significantly lower in the mutant than in wild type (Fig 3A and 3B). By contrast, another nuclear-encoded chloroplast protein, PGL35 (plastoglobulin 35 kDa), was present at similar levels in wild type and the mutant (Fig 3B). Transcription levels of the genes encoding the RuBisCO subunits and PSBA were indistinguishable between the wild type and the mutant (Fig 3C). These results suggested that *aladin-1* was impaired in the accumulation of some chloroplast proteins needed for proper chloroplast function.

### The gene responsible for the *aladin-1* phenotype is *PLASTID-SPECIFIC 50S RIBOSOMAL PROTEIN 5*, which is located next to the *ALADIN* gene

**Fig 4:**
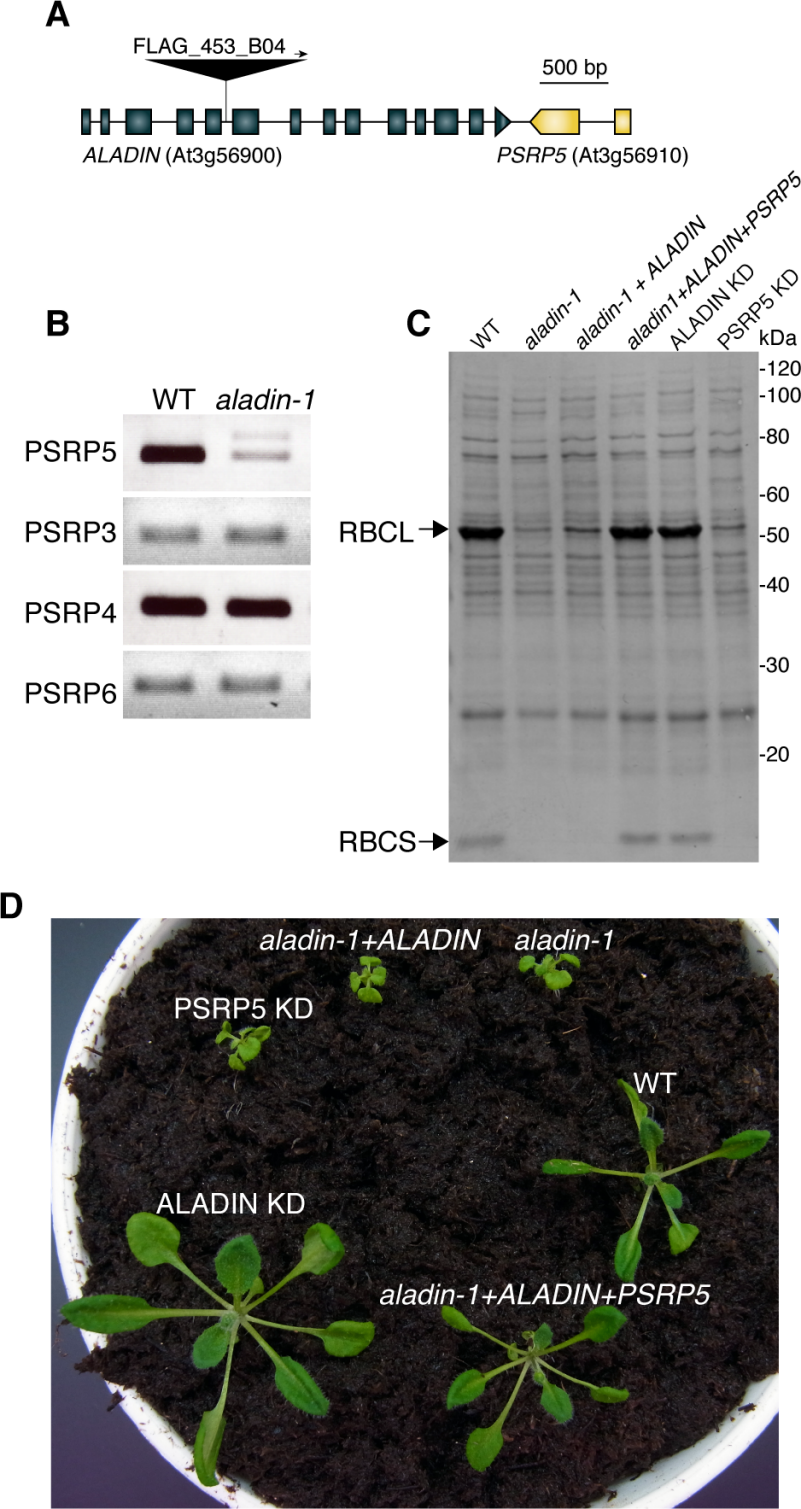
*PLASTID-SPECIFIC RIBOSOMAL PROTEIN 5* (*PSRP5*) is responsible for the *aladin-1* mutant phenotype. (A) Schematic representation of the *ALADIN* (At4g33200) and *PSRP5* (At3g56910) genes. The position of the T-DNA insertion in *aladin-1* is indicated by a triangle. Closed boxes and solid lines indicate exons and introns, respectively. (B) RT-PCR analysis of *PSRP3*, *PSRP4*, *PSRP5*, and *PSRP6* transcription in wild type (WT) and *aladin-1*. (C) Extracts of 7-day-old seedlings of WT, *aladin-1*, *aladin-1* complemented with a genomic fragment containing *ALADIN* (*aladin-1* + *ALADIN*), *aladin-1* complemented with a genomic fragment containing both *ALADIN* and *PSRP5* (*aladin-1* + *ALADIN* + *PSRP5*), aladin knockdown (ALADIN KD), and PSRP5 knockdown (PSRP5 KD) subjected to SDS-PAGE followed by Coomassie Brilliant Blue staining. Arrows indicate the positions of RBCL (upper) and RBCS (lower). (D) Three-week-old WT, *aladin-1*, *aladin-1* + *ALADIN*, *aladin-1* + *ALADIN* + *PSRP5*, ALADIN KD, and PSRP5 KD plants.

To determine whether defects in *ALADIN* were responsible for the *aladin-1* phenotype, the mutant was transformed with a genomic fragment containing wild-type *ALADIN*. However, the transformants exhibited similar RuBisCO accumulation (Fig 4C) and growth defects (Fig 4D) as *aladin-1*, indicating that the genomic fragment of *ALADIN* was unable to rescue the *aladin-1* phenotype. Transgenic plants were generated that stably expressed artificial microRNA (amiRNA) for *ALADIN* in a wild-type background (ALADIN KD plants). *ALADIN* expression was significantly lower in ALADIN KD plants than in untransformed wild-type plants (S3 Fig). However, ALADIN KD plants accumulated RuBisCO proteins normally (Fig 4C) and had a wild-type growth pattern (Fig 4D). These results indicated that *ALADIN* defects are not responsible for the mutant phenotype in *aladin-1*.

Examination of the *Arabidopsis* genome database revealed that *PLASTID-SPECIFIC RIBOSOMAL PROTEIN 5* (*PSRP5*) was located next to *ALADIN* on chromosome 3 (Fig 4A). PSRP5, which has chloroplast targeting peptide at its N-terminal, is a component of the plastid ribosomal protein (PRP) complex [24, 25]. Accumulation of *PSRP5* transcripts was significantly lower in the *aladin-1* mutant than in wild type (Fig 4B). By contrast, transcripts encoding other components of the PRP complex (*PSRP3*, *PSRP4*, and *PSRP6*) were detected at similar levels in the mutant and wild type. A genomic fragment containing *PSRP5* was able to rescue the *aladin-1* mutant defects in RuBisCO accumulation (Fig 4C) and growth (Fig 4D). Moreover, knockdown of *PSRP5* with an amiRNA construct (PSRP5 KD) (S4 Fig) phenocopied the *aladin-1* mutant defects in chloroplast proteins (Fig 4C) and growth (Fig 4D). These results indicated that *PSRP5*, rather than *ALADIN*, was responsible for the *aladin-1* phenotype.

PRPs are composed of a large 50S subunit and a small 30S subunit [25]. While the majority of PRP proteins are orthologous to bacterial proteins, plant plastids contain a small set of unique proteins termed plastid-specific ribosomal proteins (PSRPs). Several groups reported that deficiency of some PRPs and PSRPs led to pleiotropic phenotypes such as albinism, reduction of photosynthetic activity, and strongly impaired growth in *Arabidopsis* [24, 25] and rice [26]. They also reported that the T-DNA insertion line (SALK_051891) of *PSRP5* was the most-severely-affected mutant among *psrp* mutants of *Arabidopsis* [24, 25]. These results suggested that protein translation in plastids had a fundamental role in plant growth. The *aladin-1* mutant was severely impaired in chloroplast protein accumulation yet exhibited normal transcription (Fig 3). It is therefore possible that knockdown of *PSRP5*, which lies next to ALADIN on chromosome 3, is the cause of the *aladin-1* mutant phenotype.

It remains to be determined how the T-DNA insertion within *ALADIN* influences the neighboring *PSRP5* gene. Previous research found that T-DNA insertion lines harbored unexpectedly high frequencies of interchromosomal rearrangements [27]. In those cases, mutants contained single T-DNA inserts and segregated normally, but sequences from loci unlinked to the insertion site were found to flank the T-DNA border. This was not the case for *aladin-1*, in which the genomic sequence between the T-DNA border and *PSRP5* was identical to that of the corresponding wild-type sequence (S1 Fig). It is, therefore, unlikely that T-DNA integration in *aladin-1* caused interchromosomal rearrangements affecting *PSRP5* expression. However, we cannot exclude the possibility that genetic alterations at different loci from the region we sequenced affect *PSRP5* expression. Alternatively, despite the fact that the insertion occurred 2.2 kbp downstream of *PSRP5*, the T-DNA may have disrupted a cis-regulatory element controlling *PSRP5* expression (Fig 4A). Isolated regulatory regions located from their target genes have been described previously, but were mainly found in animals [28, 29]. One plant example is the *Arabidopsis GL1* gene, in which an enhancer region essential for *GL1* function is located approximately 1 kbp from the 3′ end of the coding region [30].

The nucleoporin ALADIN is thought to be an outer-ring component of the Nup107–160 nucleoporin complex, which is the largest subcomplex of the NPC [19]. The human Nup107–160 complex plays a critical role in NPC formation and scaffolding [31]. *Arabidopsis* mutations were reported in several genes encoding Nup107–160 subcomplex proteins. The *nup160* [32], *nup96* [32], *hos1* [33], and *gle1* [34] mutants exhibited severely impaired growth. However, in this study, knockdown of *ALADIN* produced no visibly defective phenotype (Fig 4D). These results indicated that *Arabidopsis* ALADIN was not required for NPC function under normal growth conditions. On the contrary, in human cultured cells, a point mutation in *ALADIN* leads to hypersensitivity to oxidative stress and subsequent accumulation of damaged DNA, resulting in cell death [35]. It is possible to expect that *Arabidopsis* ALADIN is involved in response to stress conditions in the cells. The *ALADIN* knockdown generated in this study will be a useful resource for determining the molecular role of ALADIN in the NPC.

In this study, we found that a non-target gene was unexpectedly affected by T-DNA knockdown of a neighboring gene. This emphasizes the importance of analyzing multiple mutant alleles to minimize the risk of phenotypic misinterpretation. However, where mutations are tightly linked, backcrossing with multiple alleles cannot always ensure successful genetic separation. In addition, cis-element regions of neighboring gene(s) can sometimes be included in genomic rescue constructs, and care must be taken when interpreting the results of complementation experiments that use such constructs. When unexpected phenotypes are observed, ascertaining the transcription levels of neighboring genes may be necessary to conclusively ascribe a mutant phenotype to loss-of-function of the gene of interest.

## Supporting Information

S1 Fig Genomic DNA sequence of *aladin-1* at the junction with the left border T-DNA sequence. Red, left border; Blue, *ALADIN*; Green, *PSRP5*.
(EPS)

S2 Fig RT-PCR analysis in *aladin-1*. (EPS)

S3 Fig RT-PCR analysis of *ALADIN*, *PSRP5*, and *ACT2* transcription in WT and independent ALADIN KD lines. (EPS)

S4 Fig RT-PCR analysis in WT and independent PSRP5 KD lines. (EPS)

S1 Table Sequences of PCR primers used in this study. (EPS)
